# Overexpression of *Constans* Homologs *CO1* and *CO2* Fails to Alter Normal Reproductive Onset and Fall Bud Set in Woody Perennial Poplar

**DOI:** 10.1371/journal.pone.0045448

**Published:** 2012-09-19

**Authors:** Chuan-Yu Hsu, Joshua P. Adams, Kyoungok No, Haiying Liang, Richard Meilan, Olga Pechanova, Abdelali Barakat, John E. Carlson, Grier P. Page, Cetin Yuceer

**Affiliations:** 1 Department of Forestry, Mississippi State University, Mississippi State, Mississippi, United States of America; 2 School of Forest Resources, University of Arkansas at Monticello, Monticello, Arkansas, United States of America; 3 Department of Genetics and Biochemistry, Clemson University, Clemson, South Carolina, United States of America; 4 Department of Forestry and Natural Resources, Purdue University, West Lafayette, Indiana, United States of America; 5 School of Forest Resources, Pennsylvania State University, University Park, Pennsylvania, United States of America; 6 Department of Bioenergy Science and Technology, Chonnam National University, Buk-Gu, Gwangju, South Korea; 7 Research Triangle Institute International, Atlanta, Georgia, United States of America; Instituto de Biología Molecular y Celular de Plantas, Spain

## Abstract

*CONSTANS* (*CO*) is an important flowering-time gene in the photoperiodic flowering pathway of annual *Arabidopsis thaliana* in which overexpression of *CO* induces early flowering, whereas mutations in *CO* cause delayed flowering. The closest homologs of *CO* in woody perennial poplar (*Populus* spp.) are *CO1* and *CO2*. A previous report [1] showed that the *CO2*/*FLOWERING LOCUS T1* (*FT1*) regulon controls the onset of reproduction in poplar, similar to what is seen with the *CO*/*FLOWERING LOCUS T* (*FT*) regulon in *Arabidopsis*. The *CO2*/*FT1* regulon was also reported to control fall bud set. Our long-term field observations show that overexpression of *CO1* and *CO2* individually or together did not alter normal reproductive onset, spring bud break, or fall dormancy in poplar, but did result in smaller trees when compared with controls. Transcripts of *CO1* and *CO2* were normally most abundant in the growing season and rhythmic within a day, peaking at dawn. Our manipulative experiments did not provide evidence for transcriptional regulation being affected by photoperiod, light intensity, temperature, or water stress when transcripts of *CO1* and *CO2* were consistently measured in the morning. A genetic network analysis using overexpressing trees, microarrays, and computation demonstrated that a majority of functionally known genes downstream of *CO1* and *CO2* are associated with metabolic processes, which could explain their effect on tree size. In conclusion, the function of *CO1* and *CO2* in poplar does not appear to overlap with that of *CO* from *Arabidopsis*, nor do our data support the involvement of *CO1* and *CO2* in spring bud break or fall bud set.

## Introduction

The *CONSTANS* (*CO*) gene encodes a zinc finger transcription factor that plays a major role in the photoperiodic flowering pathway of the annual and facultative long-day plant *Arabidopsis thaliana* [2]. Mutations in *CO* cause delayed flowering under long days in *Arabidopsis*, but do not affect flowering time relative to wild-type plants grown under short days, suggesting that *CO* promotes flowering under long days. *CO* transcripts are present in leaves and shoots and promote flowering in a dosage-dependent manner in *Arabidopsis*. Constitutive overexpression of *CO* from the cauliflower mosaic virus (CaMV) 35S promoter induces earlier flowering relative to wild-type plants grown under short or long days [3], [4]. *CO* transcript abundance follows a circadian rhythm, where high *CO* mRNA levels coincide with long days, but are also seen in darkness under short days [5]–[7]. CO protein is degraded in darkness by a CONSTITUTIVE PHOTOMORPHOGENIC 1 (COP1, an E3 ubiquitin ligase)-dependent mechanism when plants are grown under short days [8], but light stabilizes CO under long days through cryptochrome 2 (cry2) and phytochrome A (phyA) [9]. These mechanisms ensure the accumulation of CO protein only under long days, thus enabling flowering.

*CO* initiates flowering via upregulation of *FLOWERING LOCUS T* (*FT*) in the companion cells of leaf phloem in *Arabidopsis* [6], [10]–[15]. Following induction, the FT protein appears to be translocated through phloem to the shoot apex, where it forms a protein complex with FD (bZIP transcription factor), which upregulates *APETALA1* to initiate floral development [16]–[20].

In *Arabidopsis*, the *CO* gene family contains *CO* and 16 *CONSTANS-LIKE* (*COL*) genes within the B-box family of zinc finger proteins [21], [22]. Although *COL1* and *COL2* are under control of a circadian clock, with a peak in transcript levels at dawn, constitutive expression of *COL1* or *COL2* did not cause early- or late-flowering phenotypes in *Arabidopsis* [23], indicating that the function of these genes does not overlap with that of *CO*. The COL3 protein, which physically interacts with the COP1 protein, appears to be a positive regulator of root growth, because *col3* mutants show reduced formation of lateral roots [24]. The *col3* mutant flowers early under both long and short days, suggesting that *COL3* does not promote flowering, but may be a general repressor instead. Moreover, reduced shoot branching was observed on the *col3* mutant under short days, indicating that *COL3* regulates shoot branching in a day-length-dependent manner. *COL5* transcription is under circadian regulation and is detected in vascular tissues, but its role in controlling flowering time is unclear [25]. Constitutive expression of *COL9* resulted in plants with delayed flowering, whereas mutants with reduced *COL9* transcription flowered early under long days, suggesting that *COL9*, like *COL3*, is a floral repressor [26]. These studies show that *COL* genes are functionally unrelated to *CO*, with respect to photoperiodic flowering regulation, suggesting that they may have other roles in controlling growth and development.

*CO* homologs have been isolated from other annual or herbaceous plants such as Japanese morning glory [*Pharbitis nil* [27], [28]]; rice [*Oryza sativa* [29]]; potato [*Solanum tuberosum* ssp. *andigena* [30]]; wheat [*Triticum aestivum* [31]]; and ryegrass [*Lolium perenne* [32]]. These homologous proteins appear to be functionally conserved in photoperiodic flowering, although their coding sequences may be diverging evolutionarily across species. For example, the rice *CO* homolog (*Hd1*) inhibits transition to flowering under long days, but promotes it under short days in this short-day plant [29], [33], [34].

In woody perennials, *CO* homologs have been cloned and characterized for expression [35]–[39]. Two of 18 poplar (*Populus* spp.) *CO*-*like* genes [*CO1* (POPTR 0017s14410.1) and *CO2* (POPTR 0004s10800.1)] closely cluster with *Arabidopsis CO*, *COL1*, and *COL2* phylogenetically (see tree in Figure S1). Along with *FLOWERING LOCUS T1* (*FT1*; POPTR_0008s07730.1), *CO2* was shown to be part of a mechanism by which poplar controls reproductive onset and fall bud set by sensing critical day lengths [1]. Transcriptional repression of *CO2* via RNA interference (RNAi) in poplar appeared to cause sensitivity to short days, initiating early growth cessation and bud set [1]. If *CO2* and/or *CO1* are functionally conserved in poplar relative to *CO* in *Arabidopsis* [3], [4], their constitutive overexpression should induce early reproductive onset and delay fall bud set. To test this hypothesis, we conducted physiological and genetic experiments of *CO1* and *CO2*, including expression analysis, in poplar. Our long-term field experiments showed no evidence for involvement of these genes, singly or in combination, in reproductive onset, spring bud break, or fall bud set, suggesting that *CO1* and *CO2* in poplar are not functional orthologs of *Arabidopsis CO*.

## Results

### *CO1* and *CO2* Transcripts are Most Abundant during the Growing Season

To conduct transcript analyses reliably via quantitative reverse transcriptase polymerase chain reaction (qRT-PCR), we designed and tested gene-specific primers for *CO1* and *CO2*. The forward and reverse primers for *CO1* and *CO2* differed by five and seven nucleotides, respectively, out of 28 (Figure S2A). The primer pairs spanned the only intron present in both genes to ensure that genomic DNA, if any remained in the RNA extracts, would not be amplified. When PCR analysis was conducted using plasmid DNA harboring *CO1* or *CO2* cDNA, no cross-amplification was detected (Figure S2B). Thus, the *CO1* and *CO2* primer pairs were transcript-specific. Amplicons were cloned, sequenced, and confirmed as *CO1* and *CO2* (Figure S2B). Given that the sequence of the *CO1* and *CO2* primer binding sites diverged greatly from the remaining poplar *CO* genes (Figure S3A–B) and that the overall sequence similarity between the other members of the poplar *CO* family and *CO1* or *CO2* is low (45–57%; Figure S3C), we did not expect these primers to amplify the other poplar *CO* transcripts.

**Figure 1:**
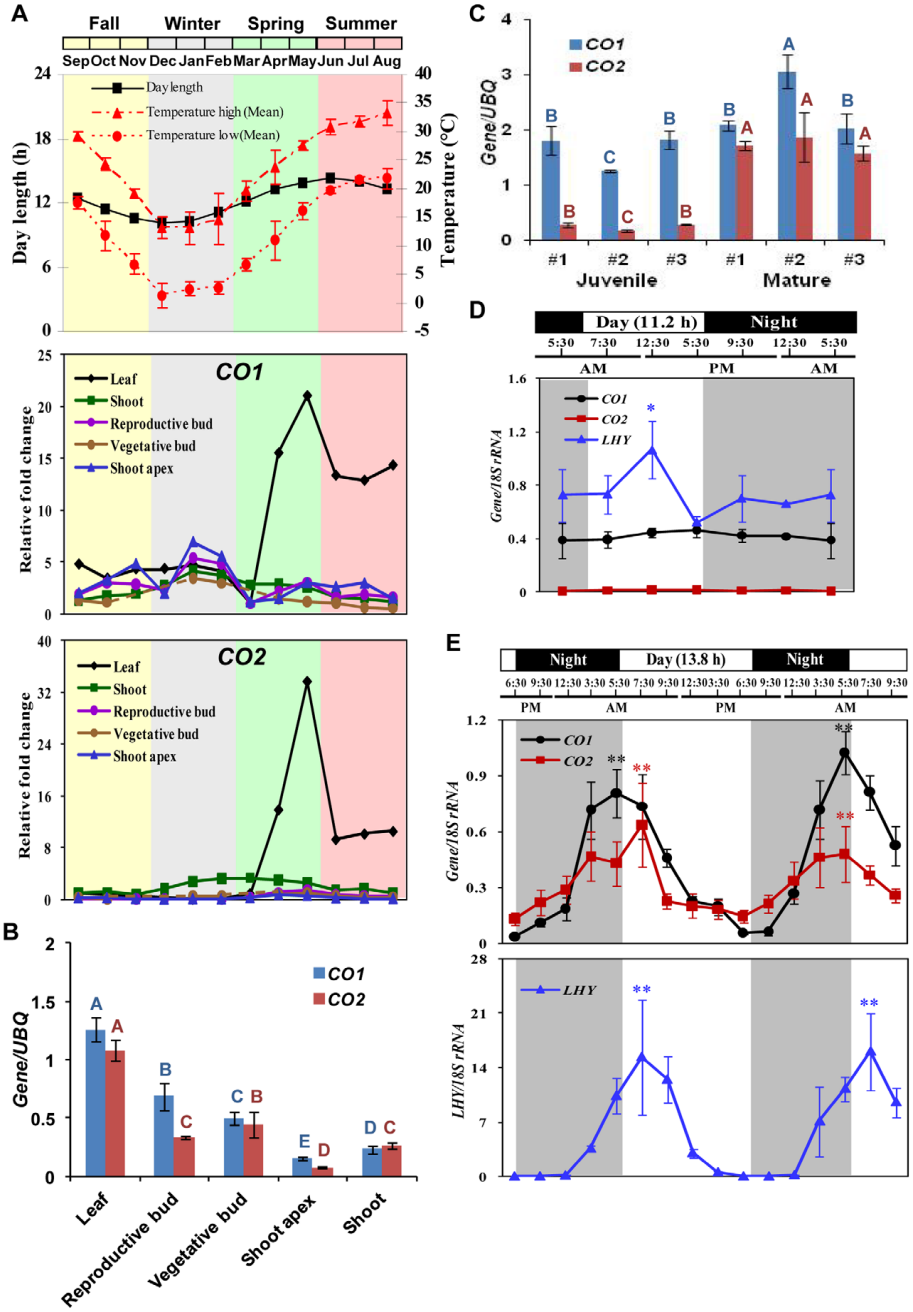
Transcript analysis of *CO1* and *CO2* via RT-PCR in field-grown *P. deltoides*. (A) Average monthly high and low temperatures and daylength in Mississippi (USA) and year-round expression of *CO1* and *CO2* in mature *P. deltoides*. *CO1* and *CO2* graphs show the relative fold change in expression levels normalized against March expression level. Dashed lines indicate missing samples. Error bars show standard deviation about the mean. (B) *CO1* and *CO2* transcripts were most abundant in leaf tissues of mature *P. deltoides* sampled in May, but least abundant in the shoot apex. Poplar *UBQ* was used as an internal control. Letters above the bars showing the abundance of *CO1* or *CO2* transcripts indicate statistically significant (*P*≤0.001) differences. Error bars indicate SD about the mean. (C) *CO1* transcripts were expressed abundantly in juvenile and mature trees (April). However, *CO2* transcripts were significantly more abundant in mature and juvenile trees. Letters above the bars showing the abundance of *CO1* or *CO2* transcripts indicate statistically significant (*P*≤0.001) differences. Error bars indicate SD about the mean. (D) Transcript abundance of *CO1* and *CO2* did not significantly (*P*>0.8) fluctuate in leaves sampled in February. *LHY* transcripts were significantly (*P*≤0.05) abundant at mid-day in the same tissues. (E) Transcript levels of *CO1* and *CO2* were significantly (*P*≤0.001) higher at dawn in leaves sampled in May, whereas *LHY* was significantly (*P*≤0.001) more abundant in the morning. *≤0.05 and **≤0.005, statistical significance within time points.

To identify the temporal and spatial expression patterns of *CO1* and *CO2*, we conducted year-round transcript analyses in various tissues of field-grown, wild-type *Populus deltoides*. Although *CO1* was expressed throughout the year at low levels in all five tissues analyzed, its transcripts were most abundant in leaves during the growing season (Figure 1A–B). *CO2* was expressed abundantly in leaves in the growing season, but at background levels at other times and in other tissues (Figure 1A–B). Although *CO1* transcripts were abundant in leaves of both juvenile and mature trees, *CO2* transcripts were significantly (*P*≤0.001) more abundant in leaves of mature trees than juveniles during the growing season (Figure 1C).

To determine whether expression of *CO1* and *CO2* fluctuates daily, we analyzed their transcripts in field-grown, wild-type *P. deltoides* at and after dawn and dusk, and in the middle of the day and the night. When *CO1* and *CO2* transcripts were analyzed in preformed leaves in February, no significant (*P*>0.8) differences were found among the six time points analyzed (Figure 1D). In contrast, we detected a significant (*P*≤0.05) difference in transcript abundance of the positive-control *LATE ELONGATED HYPOCOTYL* (*LHY*) gene at midday in the same samples (Figure 1D). In *Arabidopsis* [40] and poplar [41], *LHY* shows a circadian expression pattern with a peak in the morning under long days. Conversely, *CO1* and *CO2* expression showed a rhythm with a periodicity of about 24 h when their transcripts were analyzed in leaves in May. We detected significant differences (*P*≤0.005) among the 16 time points analyzed over 48 h (Figure 1E). *CO1* and *CO2* transcripts were significantly (*P*≤0.001) more abundant at 5∶30 AM (dawn) than at 6∶30 PM (dusk), whereas *LHY* transcripts were significantly (*P*≤0.001) more abundant at 7∶30 AM (Figure 1E).

**Figure 2:**
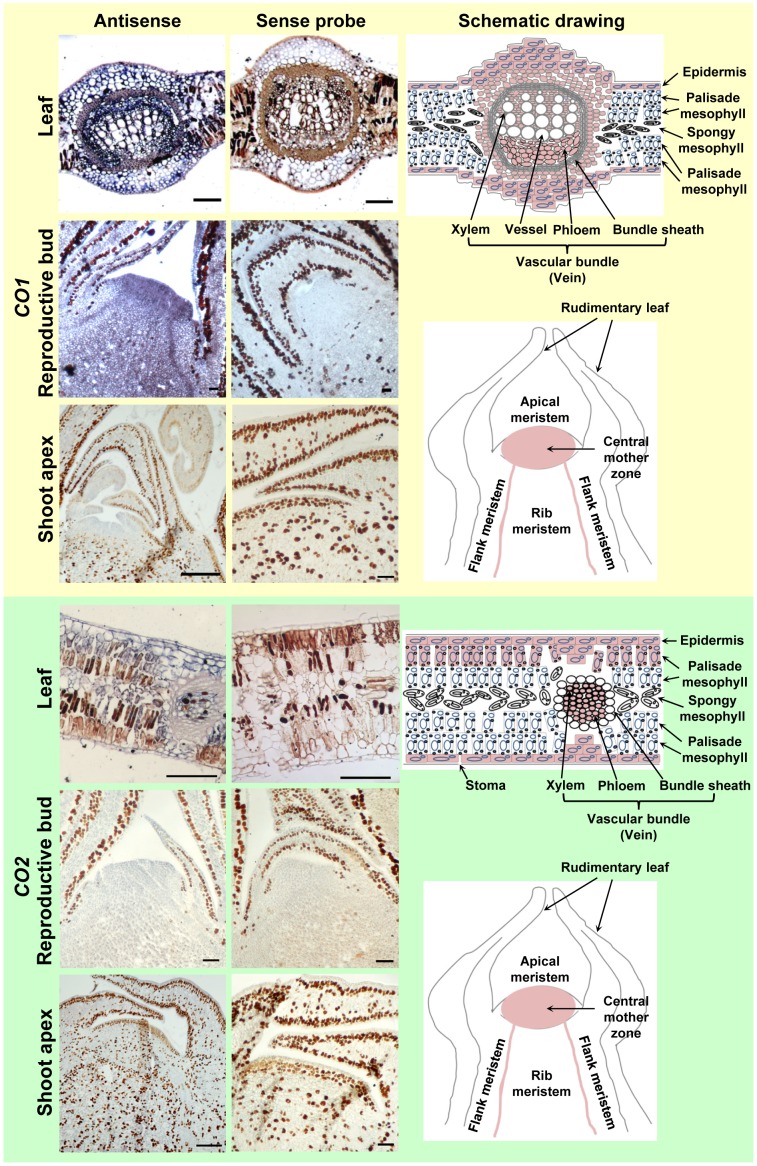
*In situ* expression analysis of *CO1* and *CO2* in leaf, reproductive bud, and shoot apex collected during the growing season from mature *P. deltoides*. Panels in the first two columns were results from the bright-field image of *in situ* hybridization and colorimetric detection of *CO1* or *CO2* transcripts. The antisense probe generated positive signals (dark blue) if present, while the sense probe served as negative control. The third column (schematic drawing) illustrates leaf cross-sections and longitudinal reproductive bud and shoot apex sections where *CO1* and *CO2* transcripts (pink color) were located, based on visual observations, as well as captured images. Scale bar, 100 µm.

To define where *CO1* and *CO2* transcripts were expressed, we conducted *in situ* expression analysis using leaves, reproductive buds, and shoot apices from mature *P. deltoides* (Figure 2). In leaf tissue, *CO1* transcripts were predominantly detected in epidermal, xylem, and phloem cells, as well as in the cells surrounding the vascular bundle. They were not detected in palisade and spongy parenchyma cells. *CO1* expression was also largely located in the apical meristem and vasculature of reproductive buds, as well as in the apical meristem and primordial (rudimentary) leaves of the shoot apex. *CO2* showed similar expression patterns to *CO1*, except that *CO2* was expressed uniformly throughout the shoot apex. Taken together, these results suggest that transcripts of *CO1* and *CO2* are most abundant in leaves and show a similar expression rhythm with a peak in transcript levels at dawn during the growing season. Their expression is predominantly confined to epidermal and vascular cells in leaves.

### *CO1* and *CO2* Transcription is not Regulated by Environmental Factors

**Figure 3:**
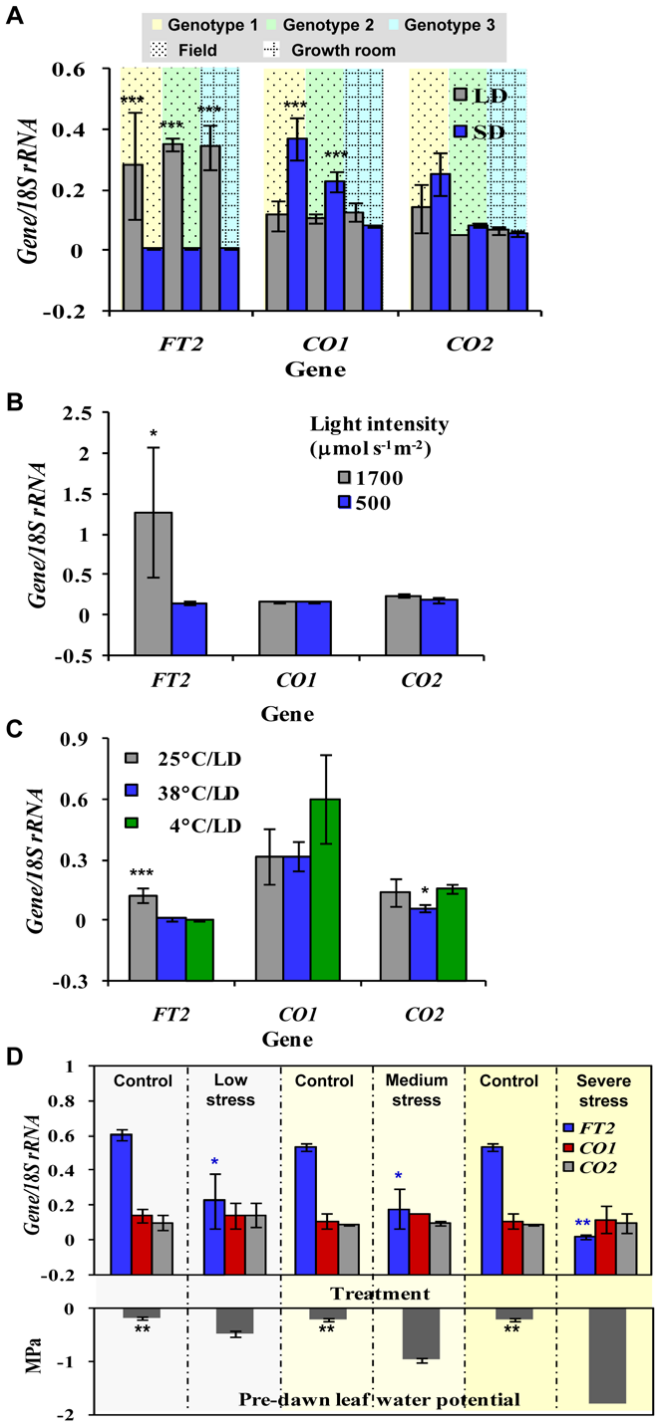
Environmental regulation of *CO1* and *CO2* transcription in mature *P. deltoides*. (A) Abundance of *CO1* transcripts increased (*P*≤0.0002) under short days (SD) in leaves of field-grown trees (two genotypes), but showed no significant (*P*>0.05) difference under SD in growth rooms. *CO2* expression did not change significantly under SD and long days (LD). *FT2* transcripts were significantly (*P*≤0.0001) less abundant under SD in trees grown both in the field and growth room. Samples were collected 2 h after sunrise or the beginning of the light period. (B) Reduced ambient light intensity did not significantly (*P*>0.05) affect *CO1* and *CO2* transcription in field-grown trees. Conversely, transcript abundance of *FT2* was significantly reduced (*P*≤0.008) at the lower light intensity. (C) Temperatures of 38, 25, and 4°C did not significantly (*P*>0.05) influence the abundance of *CO1* and *CO2* transcripts under LD, except that *CO2* transcripts were significantly (*P*≤0.05) fewer at 38°C. *FT2* transcription was significantly (*P*≤0.0005) repressed by 38°C and 4°C. (D) While *FT2* transcription decreased significantly (*P*≤0.05) under low, medium, and severe water stress (predawn leaf water potential in MPa), the levels *CO1* and *CO2* transcripts were not significantly (*P*>0.05) affected when compared with controls. *≤0.05, **≤0.005, and ***≤0.0005, statistical significance. Error bars represent standard deviation about the mean.

Because *CO1* and *CO2* are predominantly expressed in leaves during the growing season, and leaves often respond robustly to stress [42], we tested whether day length, light intensity, temperature, and water stress affect *CO1* and *CO2* transcription in leaves of mature *P. deltoides*. In the first experiment, shoots with leaves of field-grown trees were maintained under short days (8 h) and ambient long days (12–14 h) from March 25 to May 31, spanning bud break, shoot growth, and expression period of *CO1* and *CO2*. Unlike *CO2*, *CO1* transcripts were significantly (*P*≤0.0002) higher under short days (Figure 3A). However, when we repeated this experiment in a controlled environment (growth rooms) at 25°C for 42 d in spring, we did not detect a significant (*P*>0.05) difference between short and long days (Figure 3A). Transcripts of the stress-responsive *FLOWERING LOCUS T2* (*FT2*) gene [43] were significantly (*P*≤0.0001) less abundant under short days in both field and controlled experiments (Figure 3A). These results suggest that both *CO1* and *CO2* are not regulated by daylength. However, our data point to an unknown factor that regulates *CO1* in the field, but not in a controlled environment.

In the second experiment, we tested whether *CO1* and *CO2* transcription would respond to a change in light intensity. A reduction in the ambient light intensity from 1,700 to 500 µmol s^−1^ m^−2^ via continuous shading of field-grown *P. deltoides* trees for 19 days in May did not significantly (*P*>0.05) alter transcript abundance of *CO1* and *CO2*, when compared with control trees (Figure 3B). Reduced light intensity, however, significantly (*P*≤0.008) decreased the abundance of *FT2* transcripts (Figure 3B). In the third experiment, we determined whether temperature stress regulates the transcription of *CO1* and *CO2*. Actively growing, mature *P. deltoides* trees were subject to 4, 25, and 38°C under long days for 14 days in May when *CO1* and *CO2* transcripts are normally abundant in leaves. The abundance of *CO1* transcripts did not significantly (*P*>0.05) differ among the three temperature regimes, whereas *CO2* transcript abundance was significantly (*P*≤0.05) lower at 38°C (Figure 3C). *FT2* transcript abundance was significantly (*P*≤0.0005) less at both 4 and 38°C than at 25°C (Figure 3C). These results suggest that heat stress represses *CO2* transcription, but not that of *CO1*. In the final experiment, we tested whether water stress influences *CO1* and *CO2* transcription. Potted rooted cuttings of *P. deltoides* actively growing under ambient conditions were subject to low (−0.48 MPa), medium (−0.95 MPa), and severe (−1.8 MPa) water stress (predawn leaf water potential) for 19 days in May. Control plants were watered regularly and had a significantly (*P*≤0.005) higher predawn leaf water potential of about −0.2 MPa (Figure 3D). Transcript abundance of *CO1* and *CO2* did not differ significantly (*P*>0.05) among any of the water-stress regimes (Figure 3D). However, *FT2* transcription was significantly (*P*≤0.05) suppressed under the stress regimes (Figure 3D). Collectively, these experiments revealed that while *CO1* transcription was increased by an unknown factor under field conditions and *CO2* transcription was repressed by heat stress, we did not observe any effects of day length, light intensity, low temperature, and water stress on the regulation of *CO1* and *CO2* transcription.

### Overexpression of *CO1* and *CO2* Failed to Alter Normal Reproductive Onset and Fall Bud Set

**Figure 4:**
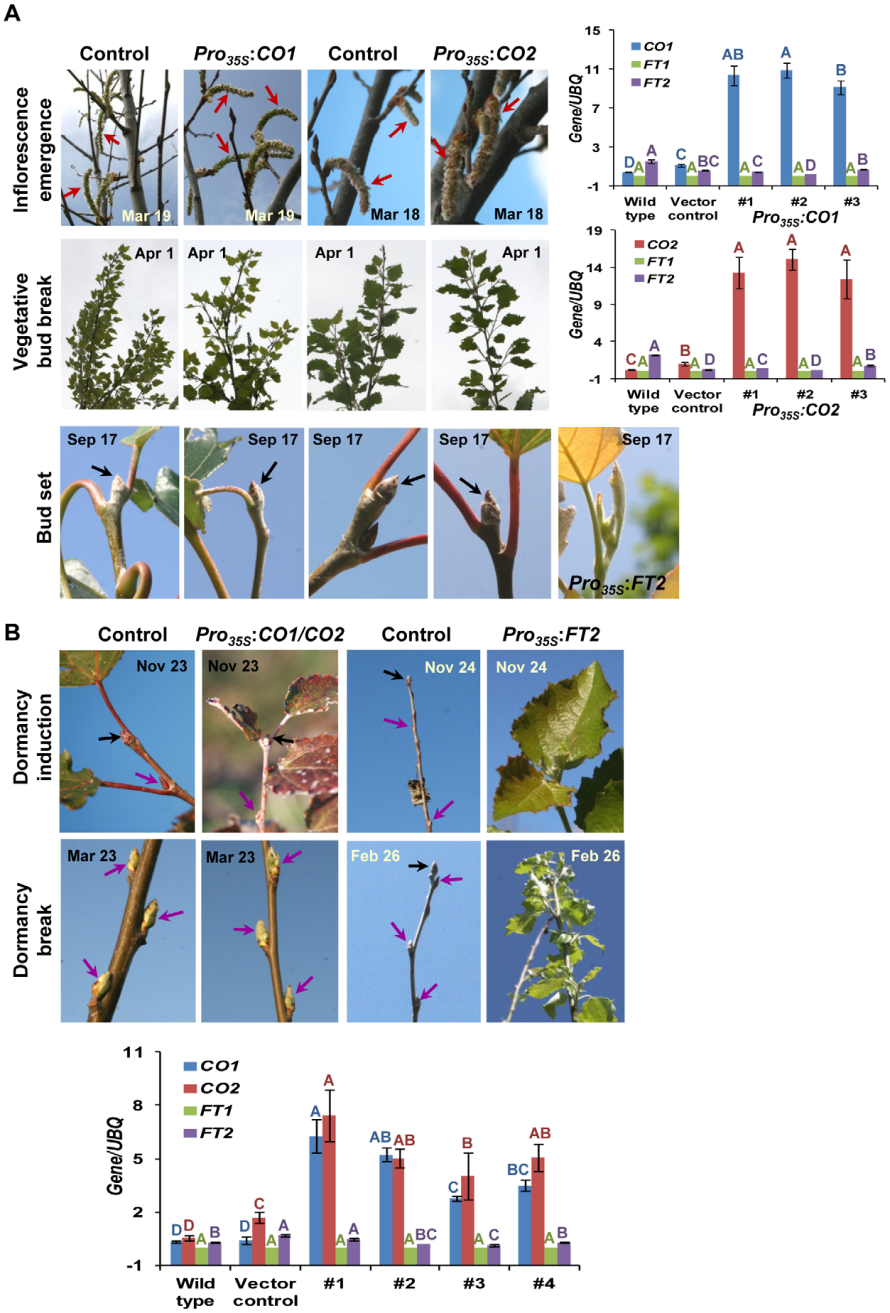
Ectopic expression of *CO1* and *CO2* individually (*Pro_35S_*:*CO1* or *Pro_35S_*:*CO2*) or together (*Pro_35S_*:*CO1/CO2*) in poplar (*P. tremula* × *P alba*). (A) When compared with controls at age 5, *Pro_35S_*:*CO1* or *Pro_35S_*:*CO2* trees did not differ in reproductive onset, spring reproductive and vegetative bud break, and fall bud set. *Pro_35S_*:*FT2* trees showed year-round active growth. Red arrows denote the emerging inflorescence in the spring, whereas black arrows point the dormant terminal vegetative bud in the fall. Unlike wild-type and vector controls, *Pro_35S_*:*CO1* or *Pro_35S_*:*CO2* trees (1, 2, and 3) significantly overproduced *CO1* or *CO2* transcripts when analyzed via qRT-PCR in leaves sampled in April. While the expression of *FT1* was undetectable, that of *FT2* fluctuated with no clear trend in controls and *CO1*- or *CO2*-overexpressing trees. Letters above the bars showing the abundance of *CO1* or *CO2* transcripts indicate statistically significant (*P*≤0.001) differences. Error bars indicate SD about the mean. (B) When *Pro_35S_*:*CO1* and *Pro_35S_*:*CO2* were co-expressed in the same trees, no difference between the transformants and controls was observed in spring bud break and fall bud set in two years. However, *Pro_35S_*:*FT2* trees showed a non-dormant phenotype. Black arrows indicate the terminal vegetative bud, whereas purple arrows point to the axillary vegetative bud. The axillary vegetative buds were opening and preformed leaves were emerging from the control and co-expressing transgenic trees on March 23. Unlike wild-type and vector-control plants, co-expressing transgenic trees (1, 2, 3, and 4) significantly overproduced *CO1* and *CO2* transcripts in leaves sampled in April. While the expression of *FT1* was undetectable, that of *FT2* fluctuated with no clear trend in controls and *CO1*/*CO2* overexpressing trees. Letters above the bars showing the abundance of *CO1* or *CO2* transcripts indicate statistically significant (*P*≤0.001) differences. Error bars indicate SD about the mean.

Two binary vectors were used to overexpress the protein-coding regions of *CO1* and *CO2* under the control of the CaMV 35S promoter, designated *Pro_35S_*:*CO1* and *Pro_35S_*:*CO2*, respectively. These constructs were independently transformed into poplar clone 717-1B4 (*P. alba* × *P. tremula*) via an *Agrobacterium*-mediated protocol. Transformants and controls were planted in the field and observed for five years, spanning all four seasons annually and in both the juvenile and mature stages of development. The transformants significantly (*P*≤0.001) overproduced *CO1* and *CO2* transcripts (Figure 4A). However, unlike controls, no significant (*P*>0.16) difference was detected in expression of either gene between morning and night in leaves of the same transformants (Figure S3D). Both transformants and controls flowered for the first time at age 5 (Figure 4A), and anthesis occurred at a similar time in March for both. Unlike *Pro_35S_*:*FT2* trees, which did not set buds in the fall or enter dormancy as long as air temperatures stayed above freezing [43], we did not observe any difference between transformants and controls in spring bud break and fall bud set. *Pro_35S_*:*CO2* trees formed significantly (*P*≤0.05) fewer flowers per tree and had significantly (*P*≤0.05) less height and diameter growth when compared with controls at age 5 (Table 1). When we measured the same traits on a cohort of *Pro_35S_*:*CO2* trees that were transformed and regenerated separately, we found similar results (Table S1). Although *Pro_35S_*:*CO1* trees were significantly (*P*≤0.05) shorter, the number of reproductive buds per tree and diameter growth were not significantly (*P*>0.05) different between transformants and controls at age 5 (Table 1).

**Table 1:** Field-grown *Pro_35S_*:*CO1*, *Pro_35S_*:*CO2*, and control trees were observed for the onset of reproduction for five years, evaluated for the number of reproductive buds or catkins at age 5, and measured for height, diameter, and shoot growth at age 5.

	*CO1*		
		Control	*Pro_35S_*:*CO1*
Anthesis	Age	5	5
	*n*	15	55
# of reproductive buds	Count	30.4 ^A^	43.5 ^A^
	*n*	15	55
Height	m	9.08 ^A^	6.95 ^B^
	*n*	15	55
Diameter	cm	6.05 ^A^	5.72 ^A^
	*n*	15	55
Shoot length	cm	39.54 ^A^	28.74 ^B^
	*n* (tree)	11	61
	*n* (total shoots)	294	1440
	***CO2***		
		**Control**	***Pro_35S_*** **:** ***CO2***
Anthesis	Age	5	5
	*n*	14	14
# of flowers	Count	305.0 ^A^	68.43 ^B^
	*n*	14	14
Height	m	7.63 ^A^	5.39 ^B^
	*n*	13	14
Diameter	cm	9.71 ^A^	4.64 ^B^
	*n*	13	14
Shoot length	cm	22.51 ^A^	24.63 ^A^
	*n* (tree)	14	14
	*n* (total shoots)	783	835

Differing letters to the right of the mean (superscript) within a row represent a statistical difference (*P*≤0.05) between the average control and average transformant. Height was measured in meter (m), whereas diameter and shoot length were measured in centimeter (cm).

Because mature trees showed abundant expression of both *CO1* and *CO2* in leaves in the growing season (Figure 1C), high co-expression of *CO1* and *CO2* in the same tree may be required for normal reproductive onset and fall bud set. To test this hypothesis, we co-transformed the *Pro_35S_*:*CO1* and *Pro_35S_*:*CO2* constructs into 717-1B4. Although the co-transformants significantly (*P*≤0.001) overproduced *CO1* and *CO2* transcripts, they neither had flowers nor showed any difference in spring bud break or fall bud set, when compared with controls by the end of year 2 in the field (Figure 4B). These results indicate that *CO1* and *CO2* are not involved in reproductive onset, spring bud break, and fall bud set.

### Poplar *CO1* Rescues the Late-flowering Phenotype of *Arabidopsis co-1* Mutant Plants

Because the second zinc finger region in *CO* of the *Arabidopsis co-1* mutant plant is defective [2], these plants have a late-flowering phenotype under long days. We transformed *co-1 Arabidopsis* with *Pro_35S_*:*CO1* to determine if it could restore the wild-type phenotype. Out of 15, three randomly selected transgenic lines (2, 8, and 10) with high levels of *CO1* expression were used in experiments. The *Pro_35S_*:*CO1* construct was able to rescue the late-flowering phenotype of *co-1 Arabidopsis* under long days in all three lines (Figure S4A–B). The transformants flowered significantly (*P*≤0.001) earlier and had fewer leaves than the *co-1* mutant plants, but flowered significantly (*P*≤0.001) later and had more leaves than wild-type controls. The *Pro_35S_*:*CO1* transformants and wild-type plants flowered within 23.5 and 22.7 days, respectively, whereas *co-1* mutants flowered within 29.9 days. *Pro_35S_*:*CO1* transformants and wild-type plants formed 16.7 and 15.7 leaves, respectively, at flowering, whereas the *co-1* mutants formed 25.6 leaves. We then sought to determine whether *CO1* upregulates the *Arabidopsis FT* gene (*AtFT*). We analyzed the expression of *AtFT* in wild-type, *co-1* mutant, and *Pro_35S_*:*CO1* plants at three developmental stages (4- and 6-leaf stages, and bolting). A gradual increase in the amount of *AtFT* transcript was detected in both wild-type and *co-1* mutants from the four-leaf to the bolting (flowering) stages, whereas the expression level of *AtFT* was generally high at all stages in all three *Pro_35S_*:*CO1* transgenic lines (Figure S4C). These results suggest that poplar *CO1* functions similarly to *CO* in *Arabidopsis* under long days.

### Genes Downstream of *CO1* and *CO2* are Predominantly Associated with Metabolism

**Figure 5:**
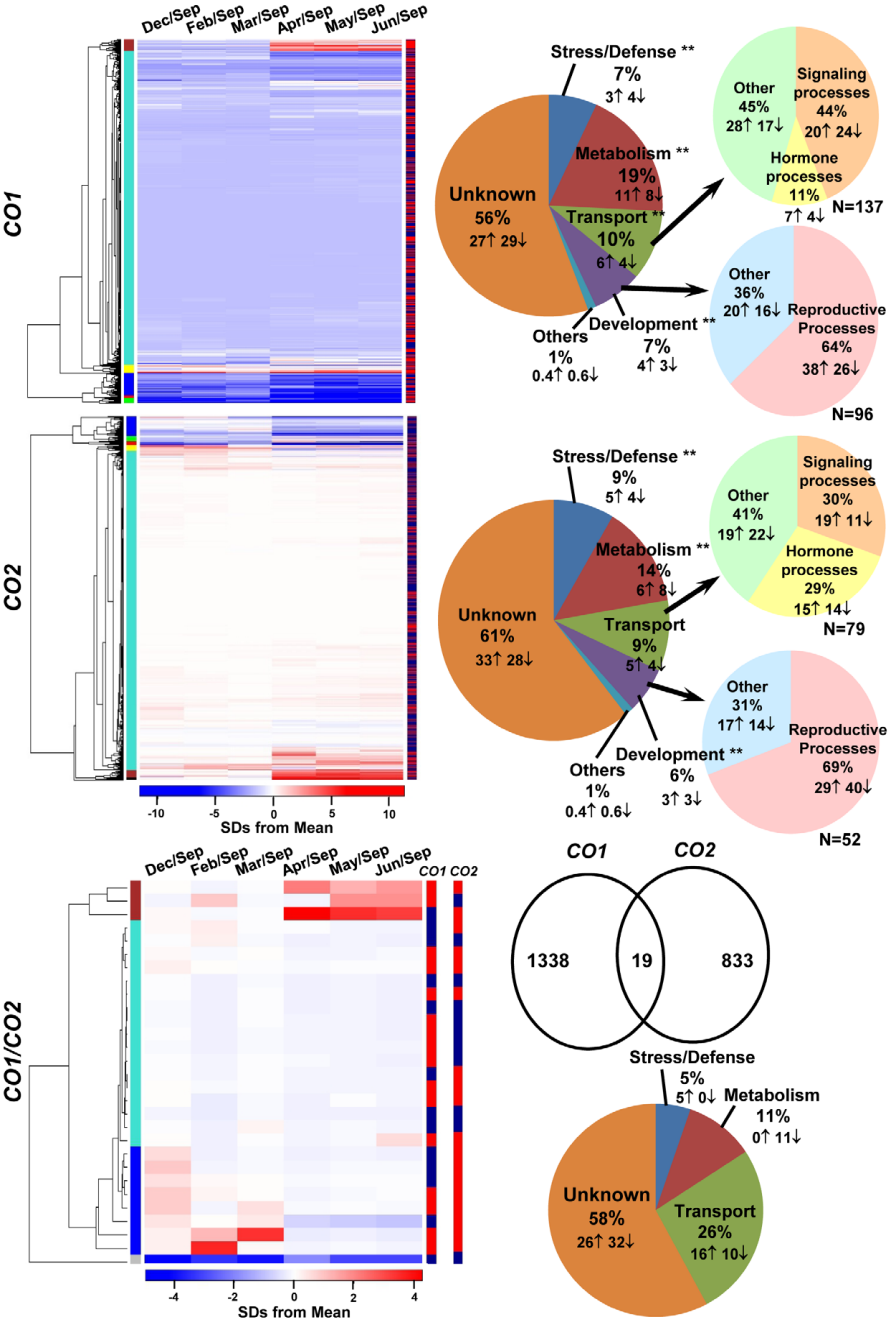
Transcripts downstream of *CO1* and *CO2* and their year-round transcript levels were identified in mature *P. deltoides* via microarray. Log_2_ fold-change of each time point relative to the baseline time point (September or Sep) was calculated. Clusters to the left of the heatmaps represent modules and the columns to right of the heatmaps represent the up- (red) and down-regulation (blue) of downstream genes. Months relative to September are above the heatmaps. The pie charts to the right of each heatmap show the functional categorization of GO Biological Process terms. N = number of genes. The Venn diagram shows the number of genes that were common to both *CO1* and *CO2* (*CO1*/*CO2*) datasets, and the pie chart below the diagram shows the GO categorization of *CO1*/*CO2* transcripts. Up (↑) and down (↓) arrows represent partitioning of overall percentage in each pie. “**” denotes the GO term is significantly (*P*≤0.001, except “development” for genes downstream of *CO1 P*≤0.006) over-represented in the microarray data when a hypergeometric test was conducted.

To determine the biological processes *CO1* and *CO2* are involved in regulating, we conducted microarray experiments comparing leaf transcript profiles of *Pro_35S_*:*CO1* and *Pro_35S_*:*CO2* trees with controls. We then evaluated year-round expression of the downstream genes of *CO1* and *CO2* using another set of previously produced microarray data from leaves of non-transgenic *P. deltoides* [43]. Cluster analysis and functional classification revealed that a considerable number of genes that act downstream of *CO1*, *CO2*, and *CO1*/*CO2* had unknown functions: 56%, 61%, and 58%, respectively (Figure 5; Tables S2, S3, S4). A majority of the downstream genes with assigned putative functions were associated with metabolism: 19% for *CO1* and 14% for *CO2*. A hypergeometric statistical test confirmed that the gene ontology (GO) term “metabolism” associated with genes downstream of *CO1* or *CO2* was significantly (*P*≤0.001) over-represented in the microarray data (Figure 5). Other, smaller groups included transport, stress/defense, and development. Although the “development” group made up a small portion of the entire list (7% of *CO1*, and 6% of *CO2*), a majority of this group was associated with reproductive processes, including genes similar to *Arabidopsis GIGANTEA* (*GI*, POPTR_0005s21870.1); FLAVIN-BINDING, KELCH REPEAT, F BOX protein 1 (*FKF1*, POPTR_0008s13460.1); circadian responses; and others that are involved in flower development. However, the GO term “reproduction” associated with genes downstream of *CO1* or *CO2* was not significantly (*P*>0.05) over-represented according to the hypergeometric test. While a small group of genes downstream of *CO1*, *CO2*, and *CO1*/*CO2* was abundantly expressed in the growing season (brown module), others often showed slight fluctuations in expression in leaves.

## Discussion

Based on phylogenetic analyses, *CO1* and *CO2* in poplar are the closest structural orthologs of *CO* in *Arabidopsis*. A previous report [1] showed that the *CO2*/*FT1* regulon controls the onset of reproduction and induction of growth cessation and bud set in poplar. This inference is partly based on observations on trees containing *CO2* RNAi constructs showing earlier than normal growth cessation and bud set when they were transferred from long to short days. It is noteworthy that the sequences for the *CO1* and *CO2* RNAi tag differed by only 9.5%. Therefore, the RNAi construct was expected to knockdown both transcripts. Based on the results, we hypothesized that increased expression of *CO1* and *CO2* should alter normal reproductive onset and bud set. However, our long-term field trials showed that overexpression of *CO1* and *CO2* singly or together in poplar does not alter normal reproductive onset, spring bud break, or fall bud set.

Recent findings of Hsu *et al*. [43] demonstrated that *FT1* expression in response to cold (e.g., 4°C) induces the onset of reproduction, as opposed to the findings by Böhlenius *et al*. [1], who showed that *FT1* is expressed and induces reproductive onset during the growing season. If the poplar *CO2*/*FT1* regulon is analogous to the *Arabidopsis CO*/*FT* in expression, regulation, and function, as Böhlenius *et al.* [1] concluded, not only should *CO2* overexpression induce the early onset of reproduction, as did *CO* [3], [4], but also *CO2* should be normally and abundantly expressed in leaves in winter along with *FT1*. However, our data show that not only did overexpression of *CO2* not induce early reproduction, but it was not expressed in winter. *CO2* was abundantly expressed in leaves only during the growing season. Although *CO1* shows a low level of expression in winter, its overexpression does not induce early or late reproductive onset in poplar. Overexpression of *CO1* was able to rescue the late-flowering phenotype of the *Arabidopsis co-1* mutant plants under long days, albeit at a slower rate than with wild-type plants, indicating that *CO1* functions somewhat similarly to *CO* in *Arabidopsis*. However, *CO1* appears not to be a strong inducer of flowering in *Arabidopsis* when compared to the overexpression of *CO* in various mutant backgrounds, such as *gi-3*, *lhy*, or *fha-1* [6], or to the overexpression of *P. nil CO* in the *co-1 Arabidopsis* [28]. Furthermore, our microarray experiments revealed that *CO1* and *CO2* in poplar are not involved in regulatory networks in the same way as *CO* in *Arabidopsis*. For example, *CO* triggers flowering via upregulation of *FT* in *Arabidopsis* leaves [6], [10]–[15], but we did not detect *FT-like* genes downstream of *CO1* or *CO2*. *GI* and *FKF1* were downstream of *CO1* and *CO2*. In the long-day *Arabidopsis* flowering pathway, GI forms a protein complex with FKF1, which then binds the *CO* promoter to regulate its transcription [44]. These observations suggest that the molecular networks controlling reproductive onset may have been modified in poplar and that poplar *CO1* and *CO2* do not appear to be functional orthologs of *Arabidopsis CO*.

What, then, are the functions of *CO1* and *CO2*? We observed that *CO1*- and *CO2*-overexpressing trees were shorter than controls. In addition, *CO2*-overexpressing trees grew less in diameter and formed fewer flowers. *CO1* and *CO2* transcripts were most abundant in leaves in the growing season and showed a diurnal rhythm that peaked at dawn, similar to the *COL1* and *COL2* circadian expression pattern in *Arabidopsis* [23]. Our physiological experiments showed that while *CO1* transcription was increased by an unknown cue under field conditions and *CO2* transcription was repressed by heat stress, other environmental factors, such as day length, light intensity, low temperature, and water stress, did not significantly regulate *CO1* or *CO2* transcription when their transcripts were consistently measured in the morning. Moreover, our microarray and computation analyses revealed that many known downstream genes of *CO1* and *CO2* are associated with metabolic processes. Based on this evidence, we hypothesize that *CO1* and *CO2* are involved in metabolic processes controlling tree size during the growing season. Perhaps, the match between daily *CO1* and *CO2* rhythms and unidentified environmental factors might contribute to this outcome. Among the functionally characterized *CO-like* genes in *Arabidopsis* and other annual plants, *COL3* in *Arabidopsis* is the only gene reported to regulate vegetative production, such as root growth and shoot branching [24]. However, a molecular mechanism has not been identified.

In conclusion, our long-term field observations show that overexpression of two poplar structural orthologs (*CO1* and *CO2*) of *Arabidopsis CO* does not alter normal reproductive onset, spring bud break, and fall bud set in poplar. *CO* is critical to regulating reproductive onset under long days in *Arabidopsis*, but our data indicate that this pathway may have been modified in poplar following the divergence of *Arabidopsis* and poplar lineages. Given the differences in life-history traits between perennial poplar and annual *Arabidopsis*, a plausible hypothesis is that poplar either does not use *CO* function in reproductive onset, or has recruited another gene with a similar function that yet has to be discovered. The fact that *CO1*- and *CO2*-overexpressing trees are smaller in size and a majority of known downstream genes of *CO1* and *CO2* is associated with metabolic processes warrants follow-up experiments on *CO1*, *CO2*, and their downstream genes in poplar.

## Experimental Procedures

### Phylogenetic Analysis

Protein sequences of CO and 16 COLs in *Arabidopsis* were retrieved from GenBank (http://www.ncbi.nlm.nih.gov). CO homologs were found in the *P. trichocarpa* genome database (http://www.phytozome.net/poplar.php) using protein-protein BLAST with E values ≤10^−5^. Multiple alignments were conducted using ClustalX [45]. The resulting alignment was used to generate a phylogenetic neighbor-joining tree via ClustalX. TreeView [46] was used to visualize the tree. Bootstrap analysis was conducted to estimate nodal support based on 1,000 replicates.

### Transcript Analysis of *CO1* and *CO2*

The coding sequences for *CO1* and *CO2* from *P. deltoides* were aligned to select dissimilar regions for designing gene-specific primers. Primer specificity was tested via PCR using recombinant plasmid DNA containing *CO1* or *CO2* as described previously [43]. To determine the year-round expression pattern of *CO1* and *CO2*, three independent replications of leaf, shoot, shoot apex, reproductive bud, and vegetative bud tissues from a wild-type, field-grown, sexually mature male *P. deltoides* tree (30 years old) located near Starkville, MS, USA, were sampled monthly 2 h after sunrise for 12 months. Due to the limited amount of tissue, we pooled the shoot apices into one sample from three replications. Total RNA was isolated as described by Wan and Wilkins [47], which was followed by DNase I digestion and cleanup using the RNeasy Mini Kit (Qiagen; Valencia, CA). Transcript abundance of *CO1* and *CO2* was analyzed by quantitative real-time (qRT)-PCR using a previously described protocol [43], the Power SYBR Green PCR Master Mix Kit (Applied Biosystems; Foster City, CA), and the 7500 Fast Real-Time PCR system (Applied Biosystems; Foster City, CA). Three technical replicates were performed for each cDNA sample. Poplar *UBIQUITIN* (*UBQ*) and *18S* rRNA were used as internal controls. Amplicon specificity and primer-dimer formation were monitored by a dissociation curve analysis after each run. Standard curves for *CO1*, *CO2*, *UBQ*, and *18S* rRNA were generated by log [cDNA] (represented by the amount of total RNA used in PCR) versus the cycle threshold (C_T_) using a series of dilutions of first-strand cDNA for each gene. The ratio between *CO1* or *CO2* and *UBQ* or *18S* rRNA for each sample was calculated using the relative quantitative analysis method [48]. Relative fold change was calculated by normalizing each expression data point for *CO1* or *CO2* with the expression data point in March. Daily high and low temperature data for 2004 to 2008 were obtained from a nearby weather station (http://ext.msstate.edu/anr/drec/weather.cgi), and a monthly average was calculated over five years. Daylength data were obtained from SunriseSunset (http://www.sunrisesunset.com) for Starkville, MS, USA. The methodology for this section is further described by Hsu *et al*. [43].

To determine the abundance of *CO1* and *CO2* transcripts in the shoot apex, vegetative bud (bud #6), reproductive bud (bud #11), shoot, and leaf, a different sexually mature *P. deltoides* tree was sampled (three independent replicates) 2 h after sunrise in May. To determine the transcript expression of *CO1* and *CO2* in juvenile and mature *P. deltoides*, four independent replicates of leaf samples were collected in April from three one-year-old (juvenile) and three mature *P. deltoides* trees. The juvenile and mature trees were not the same genotype, although they were growing in close proximity to each other. A general linear model was used to test the differences among trees for *CO1* or *CO2* expression. Means were separated by the Fisher’s protected least significant difference procedure using SAS V9 (SAS Institute; Cary, NC).

To determine whether the abundance of *CO1* and *CO2* transcripts fluctuated daily, plant material and sampling were as described by Hsu *et al.* [43], except that we used one mature *P*. *deltoides* tree with three independent samples collected at each time point. Poplar *LHY* was used as a positive control [40], [41], [43]. A general linear model was used to analyze the differences among time points via SAS. Means were separated by the Fisher’s protected least significant difference procedure.

### *In situ* Hybridization

Transcript expression of *CO1* and *CO2* was determined in the leaf, reproductive bud, and shoot apex. Samples were collected from the same mature tree mentioned above on August 8, 2005 (leaf for *CO1*), on May 15, 2006 (reproductive bud and shoot apex for *CO1* and *CO2*), and on June 17, 2005 (leaf for *CO2*). Fixation, dehydration, and clearing of samples were performed according to Jackson [49] with modifications as described in Zhang *et al*. [50]. Ten-micron sections were sliced with a microtome. Three repeats of the unique sequence from 5′ untranslated region and the beginning of the coding sequence (Table S5) were first PCR-amplified from *CO1* or *CO2* cDNAs and cloned in tandem into pGEM-T Easy (Promega; Madison, WI). *In vitro* transcription was conducted with T7 and SP6 RNA polymerase to generate sense or anti-sense mRNA, which was labeled with the DIG RNA Labeling Kit (Roche Diagnostics; Indianapolis, IN) by following the manufacturer’s instructions. Hybridization and detection steps were performed according to Drews *et al*. [51] and Zhang *et al*. [50]. For each tissue type and collection time, multiple sections from at least three samples were used for hybridization. Images were taken with an Olympus BX-60 Epi-Fluorescence Microscope equipped with Hamamatsu Orca-100 digital camera.

We did not conduct a cross-reactivity assay for *CO1* and *CO2* probes in this experiment, because the nucleotide similarity between *CO1* and *CO2* probes is relatively low (69%). The nucleotide similarity between *CO1* or *CO2* transcripts and other *CO*-like transcripts in the poplar genome is also low, ranging from 45% to 57% (Figure S3C). Thus, we expected our probes to be specific for *CO1* and *CO2* transcripts.

### Regulation of *CO1* and *CO2* Transcription

Daylength, light intensity, temperature, and water stress experiments were performed as described by Hsu *et al*. [43]. Sample collections were conducted 2 h after sunrise (field) or the beginning of the light period (growth room). Transcript analysis via qRT-PCR was conducted as described above. Poplar *FT2* was used as a control in PCR assays because of its involvement in multiple stresses [43]. A general linear model was used to analyze the effect of day length, shade, temperature, or water stress treatment on expression using SAS, and means were separated by the Fisher’s protected least significant difference procedure.

### Genetic Manipulation of *CO1* and *CO2* in Poplar

To determine whether overexpression of *CO1* and *CO2* alters normal reproductive onset and fall bud set in poplar, the overexpression vectors *Pro_35S_*:*CO1* and *Pro_35S_*:*CO2* were constructed. Coding regions of *CO1* and *CO2* were cloned into the pBI121 binary vector (BD Biosciences; Mountain View, CA) under control of the CaMV 35S promoter. The constructs were individually transformed into 717-1B4 via *Agrobacterium tumefaciens* (strain C58) [52]. The same poplar clone was also co-transformed with both binary constructs. For co-transformation, the CaMV 35S promoter and coding region of *CO1* were cloned into the pCAMBIA1300 binary vector (CAMBIA; Canberra, Australia), which contained hygromycin as a selectable marker. The *CO2*-containing binary vector was in the pBI121 backbone. The transformants carrying *Pro_35S_*:*CO1* (19 independent lines), *Pro_35S_*:*CO2* (9 independent lines), or *Pro_35S_*:*CO1*/*CO2* (7 independent lines) were planted along with controls (pBI101 empty-vector or wild-type) at the same developmental stage in the field in a completely randomized design. Each independent line was represented with at least two trees (ramets); and the total number of trees for each construct is provided in Table 1 and Table S1. Transcript abundance of *CO1*, *CO2*, *FT1*, and *FT2* was assessed via qRT-PCR in leaf tissues of wild-type, vector-control, *Pro_35s_*:*CO1*, and *Pro_35s_*:*CO2* trees. Tissues were collected 2 h after sunrise in late April. *UBQ* was used as an internal control. A general linear model was used to test the differences among trees for *CO1*, *CO2*, *FT1*, and *FT2* expression. Means were separated by the Fisher’s protected least significant difference procedure using SAS.

At age 5, *Pro_35_s*:*CO1*, *Pro_35_s*:*CO2*, and control trees were measured for height, diameter, and shoot length. Diameter was measured with a diameter tape at 1 m above ground level. Height was measured with an extendable pole. The first four primary shoots were selected from the apex of the main stem, and the secondary shoots on them were measured. The number of catkins per tree was counted. A *t*-test was used to detect differences in diameter, height, shoot length, and catkin number between *Pro_35S_*:*CO1* or *Pro_35S_*:CO2 and control trees.

### Ectopic Expression of *CO1* in Mutant *Arabidopsis*

*Pro_35S_*:*CO1* was mobilized into the *Arabidopsis co-1* mutant via *A. tumefaciens* using the floral-dip method [53]. Transformants (15) were selected on ½-strength MS salts containing 50 µg/ml kanamycin. The T_3_ generation was used for phenotypic assessment. To determine flowering time, *Pro_35S_*:*CO1* plants along with wild-type (Col-0) and *co-1* mutants were grown at 22/19°C (day/night) under long days (16 h) using 27-watt electronic fluorescent flood lights at 115 µmol s^−1^ m^−2^. Plants were arranged in a randomized complete block design with four blocks. Each genotype within a block was represented by three plants, for a total of 12 plants per genotype. Flowering time was measured by counting the number of leaves and days from seed sowing to when an inflorescence bud was seen. Analysis of variance was performed in SAS for leaf number and number of days to determine whether significant differences among genotypes could be detected for flowering time. Means were separated by the Fisher’s protected least significant difference procedure.

Leaves from three transgenic lines (#2, #8, and #10), wild-type, and *co-1* mutants were collected at the 4-leaf, 6-leaf, and bolting stages. Sample collections were conducted in the morning (8∶00 AM) 2 h from the beginning of the light period. Total RNA was isolated as described above. Expression of *CO1* and *AtFT* was analyzed via traditional RT-PCR as previously described [54]. The *18S* rRNA transcript was used as an internal control.

### Analysis of *CO1* and *CO2* Molecular Networks

Three microarray experiments were conducted to identify the genetic networks of *CO1* and *CO2* using leaves from *Pro_35S_*:*CO1* and *Pro_35S_*:*CO2* trees. First, leaf samples were collected in May from four different poplar lines harboring *Pro_35S_*:*CO1* and four control trees containing the empty vector. Leaf samples within a line were pooled. Eight microarray chips were used for this experiment. Second, leaf samples from four *Pro_35S_*:*CO2* lines and four vector-control trees were collected as for *Pro_35S_*:*CO1* lines. Eight microarray chips were used for this experiment. Third, three independent leaf samples from the same mature *P. deltoides* described above were collected in September 2005, December 2005, February 2005, March 2005, March 2006, April 2006, May 2006, and June 2006, spanning all four seasons, as previously described [43]. Thus, 24 microarray chips were used in all. All Affymetrix GeneChip Poplar Genome Array experiments were conducted as previously described [43], and data were submitted to NCBI GEO (GSE28689 and GSE28693).

To construct clusters of transcripts downstream of *CO1* and *CO2*, probes from *CO1* and *CO2* microarray data were selected with a log_2_ expression change of at least 0.5- or 2-fold from control microarray data. The selected probes were mapped over the transcript expression from previous microarray data ([43], Experiment 3, GSE24349). The year-round data were visually represented as the log_2_ ratio of each time point relative to a baseline time point (September).

Hierarchical clustering was performed on the year-round data that were present in independent *CO1* and *CO2* datasets. Also, clustering was performed for transcripts common to both *CO1* and *CO2*. In each of the three clustering analyses (i.e., *CO1*, *CO2*, and *CO1*/*CO2*), co-expression modules were determined using the “Dynamic Hybrid” algorithm [55]. Seven modules were found using the deepSplit = 1 option for both the *CO1* and *CO2* datasets. Reanalysis of the *CO1*/*CO2* data had only 4 modules using the same deepSplit option. Mapping of Gene Ontology (GO) annotations was performed as detailed in Hsu *et al*. [43]. This mapping was also conducted on the poplar genome with *Arabidopsis* ortholog pairings as available at the *P. trichocarpa* genome database version 2.2, which includes downloadable *Arabidopsis* annotations. An R script was written to assign these annotations to the poplar genes populating the microarray chips. Following annotation assignments, the number of genes with GO terms pertinent to the four general gene classifications and three sub-classifications in Hsu *et al.* [43] (e.g., metabolism, stress, reproduction) were counted. The number of genes in each group was then analyzed for over- or under-representation in the entire significant *CO1* or *CO2* array gene sets. We used methods based on Janz *et al.* [56] in which the ‘phyper’ function in R calculated a cumulative hypergeometric distribution function and then using the Benjamini-Hochberg correction, and the ‘p.adjust’ R function on the resulting p-values.

## Supporting Information

Figure S1 **Phylogenetic analysis of CO and CO-like (COL) proteins from** ***Arabidopsis thaliana*** **and poplar (** ***Populus*** **spp.).** The amino acid sequences of zinc finger family proteins, including CO and 16 COL proteins, from *A. thaliana* (At), 18 COL proteins from *P. trichocarpa* (POPTR), and CO1 and CO2 proteins from *P. deltoides* were analyzed using ClustalX and TreeView software. The analysis showed that poplar CO1 and CO2 (or POPTR 0017s14410.1 and POPTR 0004s10800.1, respectively) are the closest homologs of *Arabidopsis* CO, COL1, and COL2 (gray-boxed). Bootstrap numbers are placed at nodes in the phylogram.
(TIF)

Figure S2 **Development and testing of gene-specific primers for analysis of** ***CO1*** **and** ***CO2*** **transcripts in poplar.** (A) Primer pairs for each transcript were designed based on alignment of nucleotide sequences of *CO1* and *CO2* cDNAs isolated from *P. deltoides*. Arrows indicate the locations of forward and reverse primers. (B) PCR amplification was conducted using the designed primer pairs and recombinant plasmids harboring *CO1* and *CO2* cDNAs. The *CO1*-specific primer pair only amplified the corresponding region of *CO1* cDNA, whereas the *CO2*-specific primer pair only amplified the corresponding region of *CO2* cDNA (the left panel with two gel images). The amplicons were cloned, sequenced, and confirmed as *CO1* and *CO2* cDNAs (the right panel with nucleotide sequences).
(TIF)

Figure S3 **Sequence similarity among 18 poplar** ***CO-like*** **transcripts and constitutive expression of transgenes (** ***CO1*** **or** ***CO2*** **) in** ***Pro_35S_*** **:** ***CO1*** **or** ***Pro_35S_*** **:** ***CO2*** **trees.** (A and B) Alignment of poplar *CO-like* transcripts in the region where *CO1* and *CO2* primers are located. (C) Percent sequence similarity between *CO1* or *CO2* and other poplar *CO* family members. (D) Transcript abundance of *CO1* in leaves of *Pro_35S_*:*CO1* trees or *CO2* in leaves of *Pro_35S_*:*CO2* trees was not significantly (*P*>0.16) different between 7∶30 AM and 9∶30 PM. However, transcripts of *CO1* and *CO2* in leaves of controls were significantly (*P*≤0.05) more abundant at 7∶30 AM than at 9∶30 PM. Different letters above the bars showing the abundance of *CO1* or *CO2* transcripts indicate statistically significant differences based on a *t* test. Error bars indicate SD about the mean.
(TIF)

Figure S4 **Ectopic expression of** ***CO1*** **in** ***A. thaliana*** **and analysis of flowering time under long days.** (A) Wild-type (Col-0) and three independent *Pro_35S_*:*CO1* lines (2, 8, and 10) in the *co*-*1* mutant background were flowered earlier than the *co*-*1* mutant plants. (B) Number of days to flowering and number of leaves at flowering significantly differed (*P*≤0.001) between *Pro_35S_*:*CO1* lines in the *co-1* mutant background and controls (Col-0 and *co*-*1* mutant plants). Different letters across the bars with the same color indicate that the genotypes significantly differ for flowering time. (C) Abundance of *CO1* and *AtFT* transcripts was analyzed via RT-PCR in wild-type (WT, Col-0), *co-1* mutant, and *Pro_35S_*:*CO1* (2, 8, and 10) plants at three developmental stages of *Arabidopsis*: 4-leaf, 6-leaf, and bolting. Numbers on the left side represent the size of amplicons in base pair (bp). The *18S* rRNA was used as an internal control to verify that similar amounts of cDNA were used in the RT-PCR reaction.
(TIF)

Table S1 **An additional cohort of field-grown** ***Pro_35S_*** **:** ***CO2*** **and control trees was observed for the onset of reproduction for five years, evaluated for the number of flowers at age 5, and measured for height, diameter, and shoot growth at age 5.** Differing letters to the right of the mean (superscript) within a row represent a statistical difference (*P*≤0.05) between the average control and average transformant. Height was measured in meter (m), whereas diameter and shoot length were measured in centimeter (cm).
(DOC)

Table S2 **List of** ***CO1, CO2,*** **and** ***CO1/CO2*** **downstream genes and their associated GO annotations.** The list is prepared to match Figure 5.
(XLS)

Table S3 **Normalized expression for all probe sets in the microarray analysis that compared** ***Pro_35S_:CO1*** **to controls.**
(XLS)

Table S4 **Normalized expression for all probe sets in the microarray analysis that compared** ***Pro_35S_*** **:** ***CO2*** **to controls.**
(XLS)

Table S5 **List of primers or probes that were used for (q)RT-PCR analyses, vector construction, or** ***in situ*** **hybridization.**
(DOC)
