# Supplementary figures and images for: Overexpression of Constans Homologs CO1 and CO2 Fails to Alter Normal Reproductive Onset and Fall Bud Set in Woody Perennial Poplar
Source: PLoS One. 2012 Sep 19;7(9):e45448. doi: 10.1371/journal.pone.0045448 (PMC3446887; doi:10.1371/journal.pone.0045448)

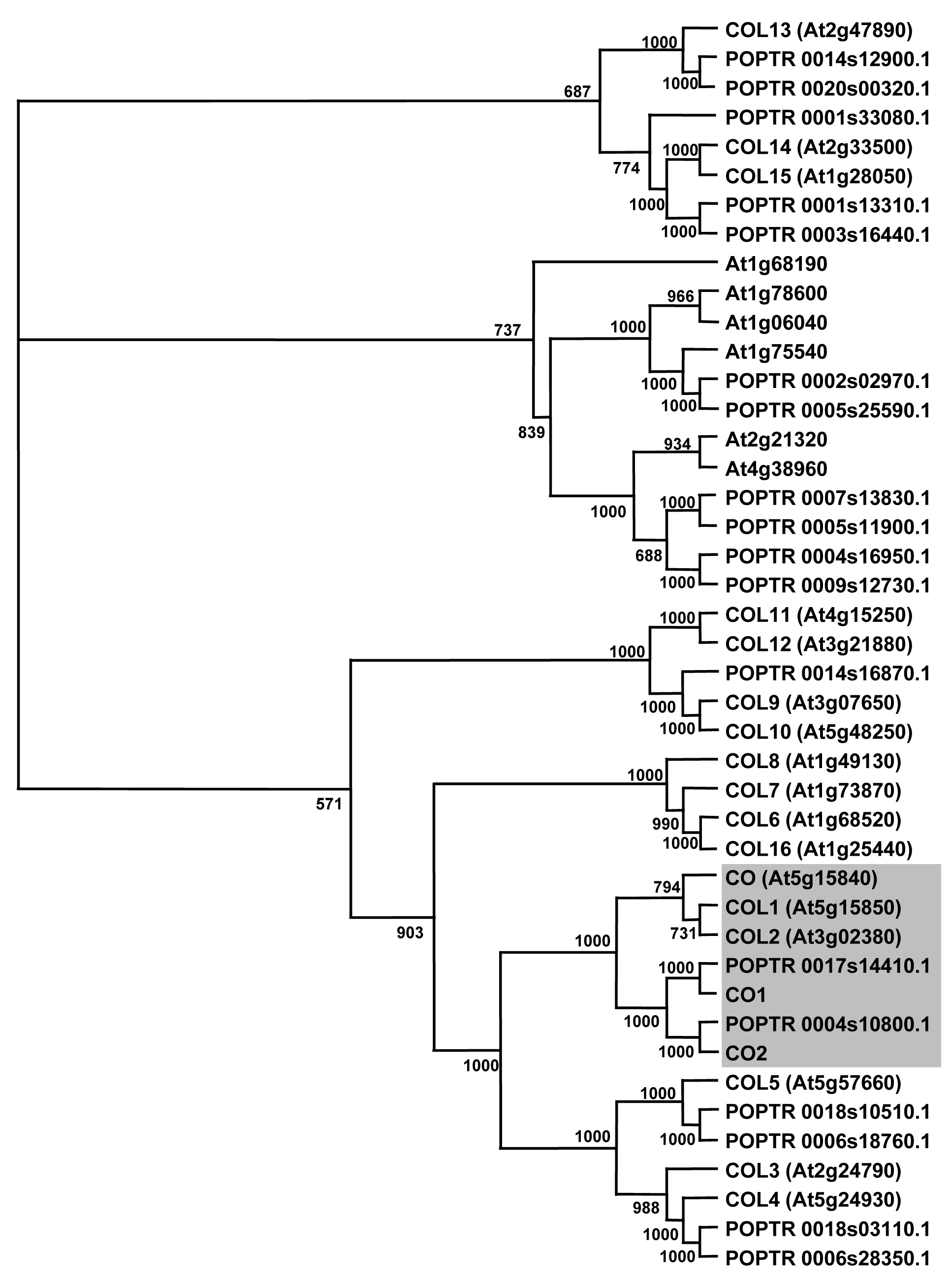

Supplement: Figure S1 — Phylogenetic analysis of CO and CO-like (COL) proteins from Arabidopsis thaliana and poplar ( Populus spp.). The amino acid sequences of zinc finger family proteins, including CO and 16 COL proteins, from A. thaliana (At), 18 COL proteins from P. trichocarpa (POPTR), and CO1 and CO2 proteins from P. deltoides were analyzed using ClustalX and TreeView software. The analysis showed that poplar CO1 and CO2 (or POPTR 0017s14410.1 and POPTR 0004s10800.1, respectively) are the closest homologs of Arabidopsis CO, COL1, and COL2 (gray-boxed). Bootstrap numbers are placed at nodes in the phylogram. (TIF) [file pone.0045448.s001.tif]

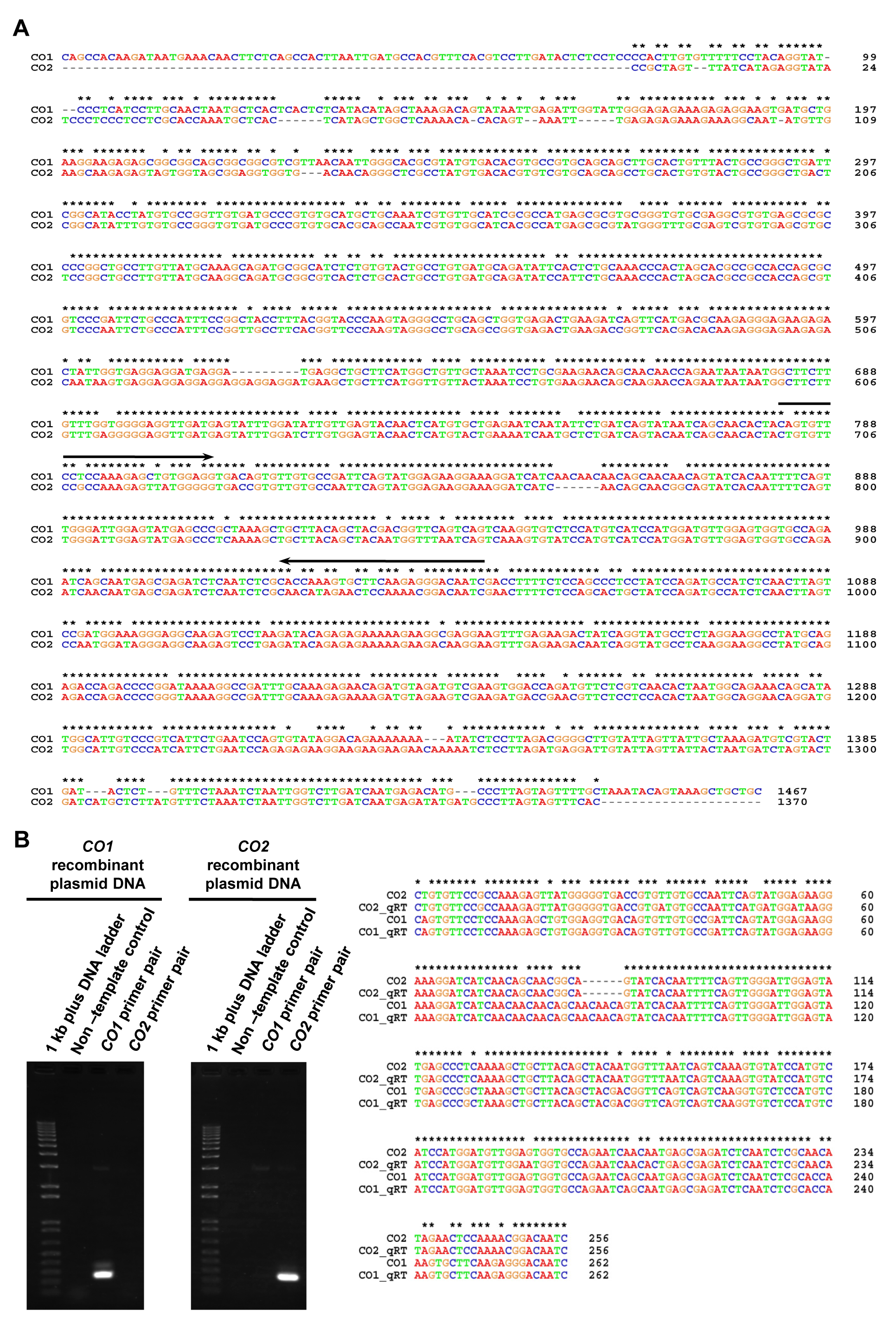

Supplement: Figure S2 — Development and testing of gene-specific primers for analysis of CO1 and CO2 transcripts in poplar. (A) Primer pairs for each transcript were designed based on alignment of nucleotide sequences of CO1 and CO2 cDNAs isolated from P. deltoides. Arrows indicate the locations of forward and reverse primers. (B) PCR amplification was conducted using the designed primer pairs and recombinant plasmids harboring CO1 and CO2 cDNAs. The CO1-specific primer pair only amplified the corresponding region of CO1 cDNA, whereas the CO2-specific primer pair only amplified the corresponding region of CO2 cDNA (the left panel with two gel images). The amplicons were cloned, sequenced, and confirmed as CO1 and CO2 cDNAs (the right panel with nucleotide sequences). (TIF) [file pone.0045448.s002.tif]

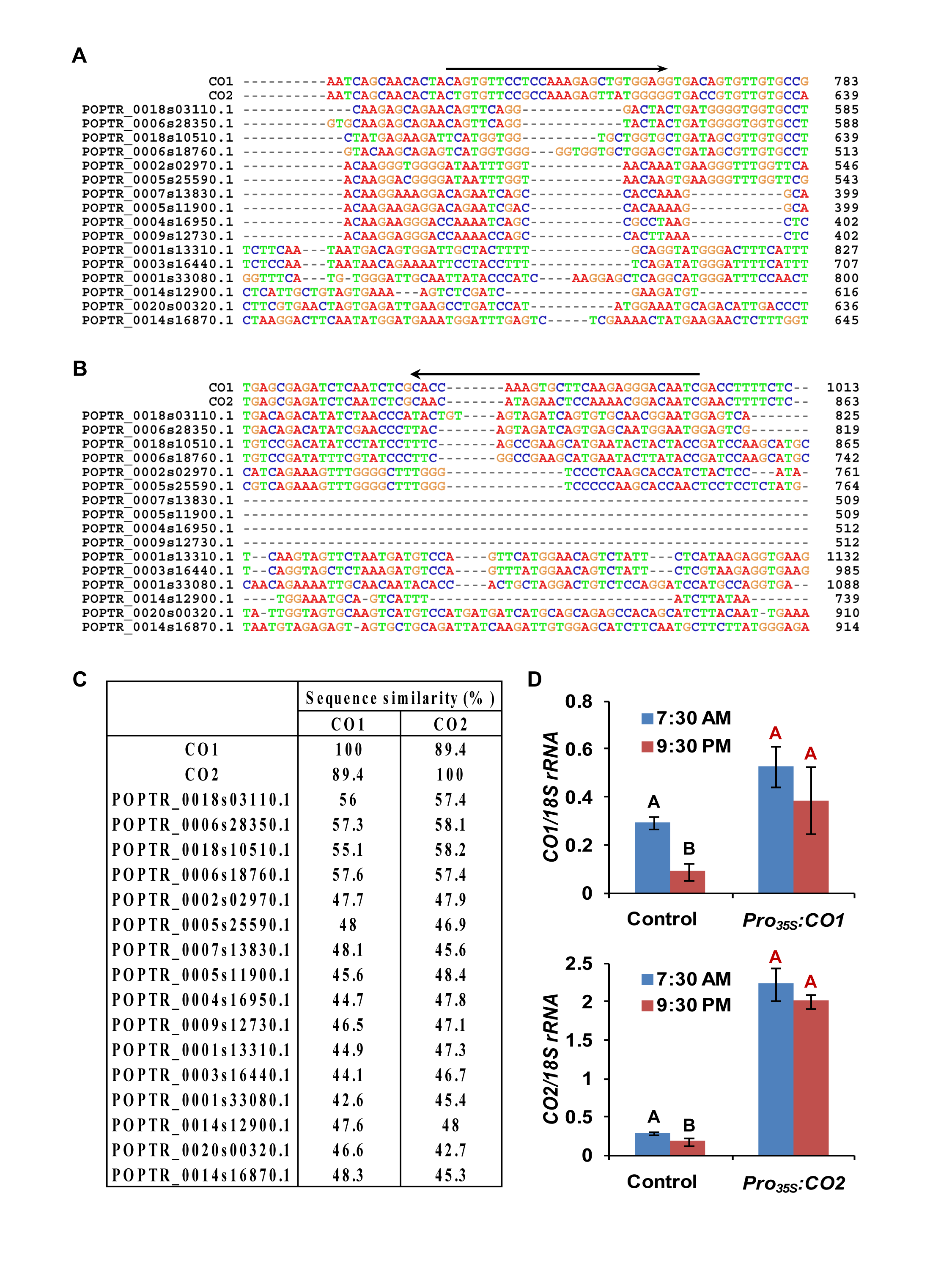

Supplement: Figure S3 — Sequence similarity among 18 poplar CO-like transcripts and constitutive expression of transgenes ( CO1 or CO2 ) in Pro35S : CO1 or Pro35S : CO2 trees. (A and B) Alignment of poplar CO-like transcripts in the region where CO1 and CO2 primers are located. (C) Percent sequence similarity between CO1 or CO2 and other poplar CO family members. (D) Transcript abundance of CO1 in leaves of Pro35S:CO1 trees or CO2 in leaves of Pro35S:CO2 trees was not significantly (P>0.16) different between 7∶30 AM and 9∶30 PM. However, transcripts of CO1 and CO2 in leaves of controls were significantly (P≤0.05) more abundant at 7∶30 AM than at 9∶30 PM. Different letters above the bars showing the abundance of CO1 or CO2 transcripts indicate statistically significant differences based on a t test. Error bars indicate SD about the mean. (TIF) [file pone.0045448.s003.tif]

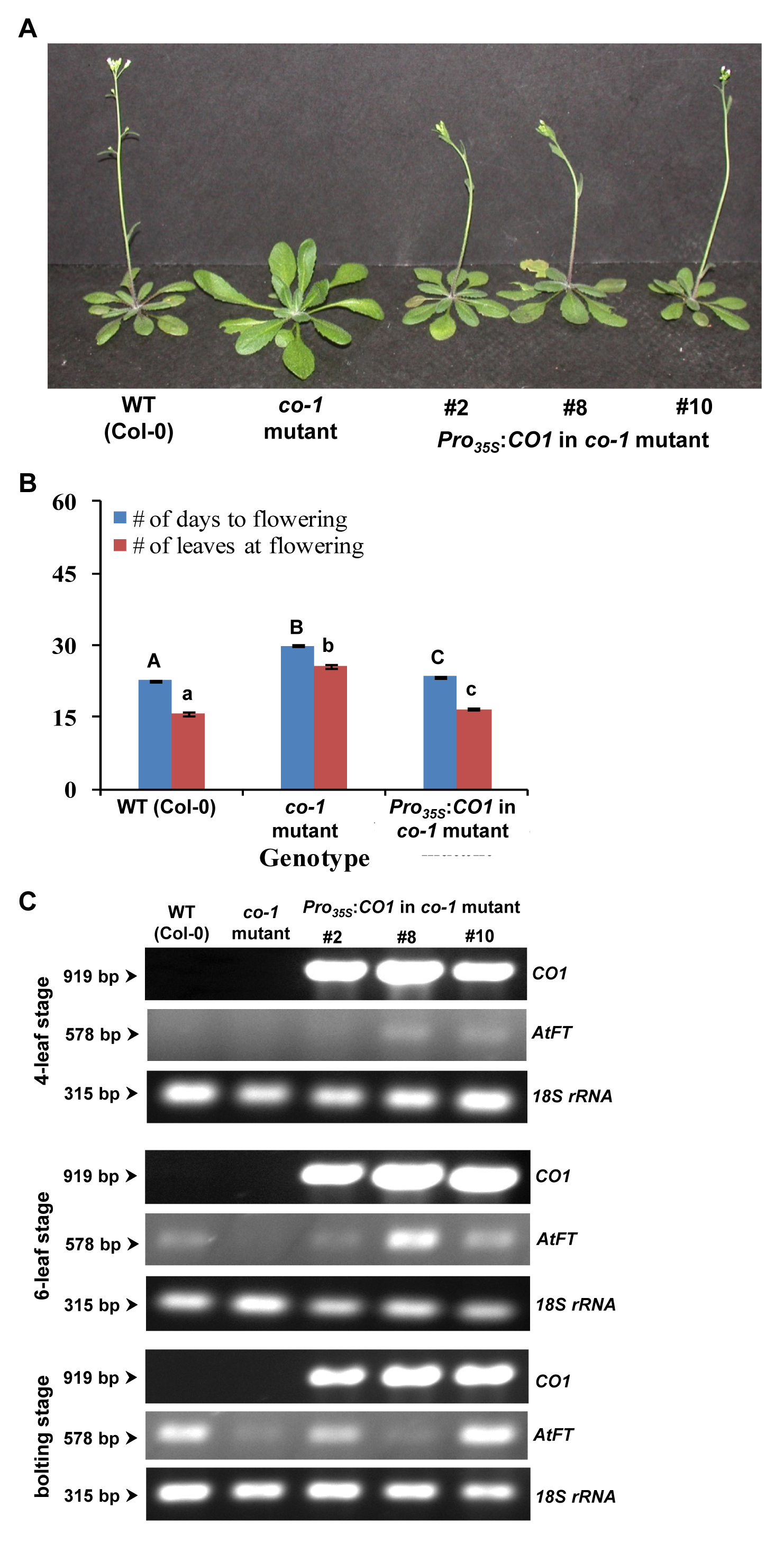

Supplement: Figure S4 — Ectopic expression of CO1 in A. thaliana and analysis of flowering time under long days. (A) Wild-type (Col-0) and three independent Pro35S:CO1 lines (2, 8, and 10) in the co-1 mutant background were flowered earlier than the co-1 mutant plants. (B) Number of days to flowering and number of leaves at flowering significantly differed (P≤0.001) between Pro35S:CO1 lines in the co-1 mutant background and controls (Col-0 and co-1 mutant plants). Different letters across the bars with the same color indicate that the genotypes significantly differ for flowering time. (C) Abundance of CO1 and AtFT transcripts was analyzed via RT-PCR in wild-type (WT, Col-0), co-1 mutant, and Pro35S:CO1 (2, 8, and 10) plants at three developmental stages of Arabidopsis: 4-leaf, 6-leaf, and bolting. Numbers on the left side represent the size of amplicons in base pair (bp). The 18S rRNA was used as an internal control to verify that similar amounts of cDNA were used in the RT-PCR reaction. (TIF) [file pone.0045448.s004.tif]
