# Supplementary material for: Overexpression of Constans Homologs CO1 and CO2 Fails to Alter Normal Reproductive Onset and Fall Bud Set in Woody Perennial Poplar
Source: PLoS One. 2012 Sep 19;7(9):e45448. doi: 10.1371/journal.pone.0045448 (PMC3446887; doi:10.1371/journal.pone.0045448)
Supplement: Table S1 — An additional cohort of field-grown Pro35S : CO2 and control trees was observed for the onset of reproduction for five years, evaluated for the number of flowers at age 5, and measured for height, diameter, and shoot growth at age 5. Differing letters to the right of the mean (superscript) within a row represent a statistical difference (P≤0.05) between the average control and average transformant. Height was measured in meter (m), whereas diameter and shoot length were measured in centimeter (cm). (DOC) [file pone.0045448.s005.doc]

Table S1.

|  |  | **Control** | ***Pro_35S_*:*CO2*** |
| --- | --- | --- | --- |
| **Anthesis** | **Age** | **5** | **5** |
|  | ***n*** | **13** | **8** |
| **# of flowers** | **Count** | **682.25 ^A^** | **453.38 ^B^** |
|  | ***n*** | **8** | **8** |
| **Height** | **m** | **7.96 ^A^** | **6.79 ^B^** |
|  | ***n*** | **13** | **8** |
| **Diameter** | **cm** | **10.02 ^A^** | **7.59 ^B^** |
|  | ***n*** | **13** | **8** |
| **Shoot length** | **cm** | **20.07 ^A^** | **21.05 ^A^** |
|  | ***n* (tree)** | **8** | **8** |
|  | ***n* (total shoots)** | **378** | **356** |
