# Supplementary material for: Overexpression of Constans Homologs CO1 and CO2 Fails to Alter Normal Reproductive Onset and Fall Bud Set in Woody Perennial Poplar
Source: PLoS One. 2012 Sep 19;7(9):e45448. doi: 10.1371/journal.pone.0045448 (PMC3446887; doi:10.1371/journal.pone.0045448)
Supplement: Table S5 — List of primers or probes that were used for (q)RT-PCR analyses, vector construction, or in situ hybridization. (DOC) [file pone.0045448.s009.doc]

Table S5.

| Experiments | Primer name | Sequence (5' to 3') | Amplicon size (bp) |
| --- | --- | --- | --- |
| vector construction | PdCO1-PT1 (forward) | 5’- gatctaga^1^ATGCCACGTTTCACGTCCTTG^21^ -3’ | 1269 bp |
|  | PdCO1-PT2 (reverse) | 5’- tagagctc^1269^TCAGAATGACGGGACAATGCC^1249^ -3’ |  |
|  | PdCO2-PT1 (forward) | 5’- ggtctaga^1^ATGTTGAAGCAAGAGAGTAG^20^ -3’ | 1119 bp |
|  | PdCO2-PT2 (reverse) | 5’- ccggatcc^1119^TCAGAATGATGGGACAATGCC^1099^ -3’ |  |
|  | * Lower-case letters represent the restriction enzyme digestion sites: | |  |
|  | XbaI (tctaga), BamHI (ggatcc), and SacI (gagctc) | |  |
|  |  |  |  |
| RT-(q)PCR | PdCO1-RT1 (forward) | 5’-^741^CAGTGTTCCTCCAAAGAGCTGTGGAG^766^-3’ | 262 bp |
|  | PdCO1-RT2 (reverse) | 5’-^1002^GATTGTCCCTCTTGAAGCACTTTGGTG^976^-3’ |  |
|  | PdCO2-RT1 (forward) | 5’-^597^CTGTGTTCCGCCAAAGAGTTATGGGG^662^-3’ | 256 bp |
|  | PdCO2-RT2 (reverse) | 5’-^852^GATTGTCCGTTTTGGAGTTCTATGTTG^826^-3’ |  |
|  | UBQ-1 (forward) | 5’-^525^CGATAATGTGAAGGCCAAAATTCAG^549^-3’ | 278 bp |
|  | UBQ-2 (reverse) | 5’-^802^GGTCAGGGGGTATTCCTTCCTTGTC^778^-3’ |  |
|  | 18S rRNA-1 (forward) | 5’-GGAATTGACGGAAGGGCACCACCAGGC-3’ | 315 bp |
|  | 18S rRNA-2 (reverse) | 5’-GGACATCTAAGGGCATCACAGACCTG-3’ |  |
|  | AtFT-5E1 (forward) | 5’-^-24^CCACCTGTTTGTTCAAGATCAAAG^-1^-3’ | 578 bp |
|  | AtFT-3E2 (reverse) | 5’-^554^CAATTGGTTATAAAGGAAGAAGCC^531^-3’ |  |
|  | FT1-RT1 (forward) | 5'-^284^CAACTGGGGCAAGCTTTGGCCATGAAAC^311^-3' | 242 bp |
|  | FT1-4 (reverse) | 5'-^525^TTATCGCCTCCTACCACCAGAGCCAC^500^-3' |  |
|  | FT2-RT1 (forward) | 5'-^284^CTACCGGGGCGAACTTTGGGCAAGAGG^310^-3' | 242 bp |
|  | FT2-2 (reverse) | 5'-^525^TCATGGTCTCCTTCCACCGGAGCCAC^500^-3' |  |

Table S5. continued

| *In* *situ* hybridization | Probe | Sequence (5' to 3') |  |
| --- | --- | --- | --- |
|  | *CO1* | 5'-cagccacaagataatgaaacaacttctcagccacttaattgatgccacgtttcacgtccttgatactctcctccccacttgtgtttttcctacaggtatccctcatccttgcaactaatgctcactcactctcatacatagctaaagacagtataattgagattggtattgggagagaaagagaggaagtgatgctgaaggaagagagcggcggcagcggcggcgtcgttaacaat-3’ |  |
|  | *CO2* | 5'-ccgctagtttatcatagaggtatatccctccctcctcgcaccaaatgctcactcatagctggctcaaaacacacagtaaatttgagagagaaagaaaggcaatatgttgaagcaagagagtagtggtagcggaggtggtgacaacagggctcgcctatgtga-3’ |  |
